# Optimizing strategy for the discovery of compositionally-biased or low-complexity regions in proteins

**DOI:** 10.1038/s41598-023-50991-8

**Published:** 2024-01-05

**Authors:** Paul M. Harrison

**Affiliations:** Department of Biology, McGill University, Montreal, QC Canada

**Keywords:** Computational biology and bioinformatics, Protein analysis, Sequence annotation

## Abstract

Proteins can contain tracts dominated by a subset of amino acids and that have a functional significance. These are often termed ‘low-complexity regions’ (LCRs) or ‘compositionally-biased regions’ (CBRs). However, a wide spectrum of compositional bias is possible, and program parameters used to annotate these regions are often arbitrarily chosen. Also, investigators are sometimes interested in longer regions, or sometimes very short ones. Here, two programs for annotating LCRs/CBRs, namely SEG and fLPS, are investigated in detail across the whole expanse of their parameter spaces. In doing so, boundary behaviours are resolved that are used to derive an optimized systematic strategy for annotating LCRs/CBRs. Sets of parameters that progressively annotate or ‘cover’ more of protein sequence space and are optimized for a given target length have been derived. This progressive annotation can be applied to discern the biological relevance of CBRs, e.g., in parsing domains for experimental constructs and in generating hypotheses. It is also useful for picking out candidate regions of interest of a given target length and bias signature, and for assessing the parameter dependence of annotations. This latter application is demonstrated for a set of human intrinsically-disordered proteins associated with cancer.

## Introduction

Despite being composed of an alphabet of twenty diverse amino acids, proteins can often demonstrate a compositional bias (CB) for a small subset of this residue alphabet. For example, the sequence PPQPPSPPPSPPPPPQPPP is biased for the single residue P (proline), and the sequence ARGGRARGARSRRGAAAGAGGRAGSAG is biased for A (alanine), R (arginine) and G (glycine). Simpler, more repetitive regions that tend to be shorter are often termed ‘low-complexity’ regions (LCRs). However, longer compositionally-biased regions (CBRs) can have quite a mild compositional skew, and LCRs can be considered a subset of CBRs. Many CBRs are composed of tandem repeats of sequence units several residues long. CBRs can be found in intrinsically disordered proteins, fibrous proteins, cell-structural proteins, functional amyloids and prions, or globular domains with specific functional roles such as metal binding^1^. They are also a significant component of the ‘dark proteome’ that has been chronically un- or understudied^2^.

Discovery of LCRs/CBRs in proteins has been actively researched, with several programs being developed. These include SIMPLE^3,4^, SEG^5,6^, 0j.py^7^, ScanCom^8^, CARD^9^, BIAS^10^, SARP^11^, LCD-Composer^12,13^ and LPS/fLPS^1,14–16^. Lee*, *et al. developed a method for picking out low-complexity regions using image processing of dot plots^17^. Furthermore, servers pooling results of multiple methods have been produced, including LCT, LCReXXXplorer and PLaToLoCo^18–20^. SEG labels LCRs by scanning sequences with a fixed window length and applying thresholds for sequence entropy^5^. This algorithm has long been a component of the BLAST sequence alignment suite, wherein it can filter for false positive sequence matches arising because of simple, low-complexity sequence composition^21^. The program fLPS uses binomial probability to pick out low-probability sequence tracts^14,15^. It has been applied to studying the evolution of prions and prion-like regions^22–26^, and to characterizing the ‘dark proteome’^2^, and by many other investigators to aid in characterization of protein sub-domains^15^.

SEG and fLPS are particularly useful for large-scale automated analysis of CBRs, since they do not require specification of residue type lists. They can characterize regions made from multiple-residue bias; they can also delineate milder biased regions. The benefits of SEG include that background amino-acid frequencies do not need to be considered, and its rapid calculations^5^. fLPS is a faster algorithm that works by detecting specific amino-acid biases^14,15^. Single-residue and multiple-residue CBRs are calculated explicitly, several options for background amino-acid frequencies are offered, and its two-window system is designed to capture a diversity of region lengths.

What is a low-complexity region and what is not? When is a protein domain compositionally biased? The answer to these questions is not simple. Many different thresholds are possible for labelling these regions that will ‘cover’ smaller or larger amounts of protein sequences. Also, investigators might be interested in very short regions, or sometimes longer ones. Although, algorithms for their discovery have been published with ‘recommended’ parameter sets for CBR annotation, there has been no systematic, thorough examination of what parameters are suitable, and how parameter choice relates to region length. Here, I examine the performance of SEG and fLPS across the whole expanse of their parameter spaces. In doing so, boundary behaviours are discovered that are used to derive an optimized strategy for annotation of LCRs or CBRs of given target lengths.

## Methods

### Data

Two sequence sets were downloaded from UniProt (uniprot.org)^27^ in June 2022. These are: (i) *Saccharomyces cerevisiae* strain S288C proteome (number UP000002311, 5879 sequences); (ii) UniRef50 representative protein set. The latter was reduced to a 0.1% random sample (52,523 sequences, i.e., every 1000th sequence). A data set of water-soluble non-membrane protein domain sequences < 40% identical to each other was generated from the ASTRAL sequence data available at SCOPe (scop.berkeley.edu)^28^. Atom record sequences were used to avoid including intrinsically disordered regions that have no electron density in crystallographic data.

A data set of 137 human intrinsically-disordered proteins (IDPs) associated by database curators with cancer was downloaded from the Uniprot database (uniprot.org) in October 2023.

### Running the SEG and fLPS programs

**Figure 1:**
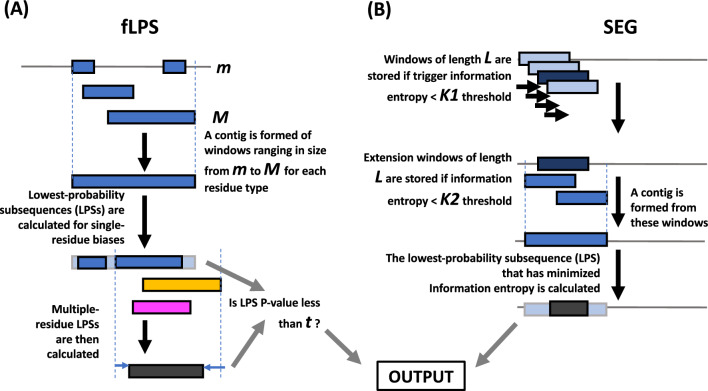
Schematics of the algorithms. (**A**) fLPS algorithm. The three main parameters are maximum and minimum window lengths **M** and **m**, and an output P-value threshold **t**. Window lengths down from **M** to **m** are searched for single-residue biases with P-values less than a fixed high threshold (= 0.001 here). These are then used to form contigs out of which lowest-probability subsequences (LPSs) are calculated (multiple LPSs from the same contig are possible). Then, multiple-residue biased regions are tested for, which includes trimming or extending to obtain the multiple-residue LPS. Finally, the output is filtered with **t**, the threshold P-value. (**B**) SEG algorithm. Regions with low sequence entropy are more ‘ordered’ since they are dominated by a few of the possible amino-acid residue types. SEG works by scanning along sequences for windows of length **L** that have sequence entropy ≤ **K1**, a trigger threshold. Then, these ‘trigger windows’ are extended with further windows whose sequence entropy is ≤ **K2**, the extension threshold, to form a contig. SEG LPSs are then calculated from these contigs using recursion.

The two programs SEG and fLPS were investigated across a thorough sample of their parameter spaces. Both programs use three main parameters for CBR discovery, but extract fundamentally different information from the sequences. Figure 1 details both algorithms. The fLPS program (Fig. 1A) works through a process of binomial probability minimization^14^. A binomial P-value can be calculated for the amino-acid biases of any sequence tract. SEG uses sequence entropy to search for CBRs/LCRs (Fig. 1B).

The parameter spaces searched are tabulated (Table 1). For fLPS, default background ‘domains’ amino-acid frequencies were used. These were updated using the downloaded ASTRAL sequences (however, the frequencies have changed minimally indicating likely convergence). For both programs, there are recommended parameter sets to discover shorter low-complexity regions (Table 1). Also, there are SEG parameter sets to label longer biased regions, such as those made of longer tandem repeats, whose repetitiveness is only captured by longer **L** windows. The default fLPS settings are intended as a ‘catch-all’ set of very loose parameters that informs users of all CBRs in their sequences, even very mild short tracts. After a default run, the user is expected to home in on biases of interest with smaller **t** P-value thresholds. For example, **t** = 1e−10 was used to study prion-like proteins, since 1e-10 was the highest P-value observed for known prion-forming protein tracts (Table 1).

**Table 1:** Parameter values analyzed for SEG and fLPS.

	Recommended parameter sets	Parameter space tested
fLPS*	*For shorter low-complexity regions:* **m** = 5, **M** = 25, **t** = 1e−05 **m** = 5, **M** = 25, **t** = 1e−06 *To find most shorter low-complexity tracts, and longer compositionally biased regions (default), i.e., ‘catch-all’ default parameters:* **m** = 15, **M** = 500, **t** = 1e-03 *Parameters used for identifying prion-like compositional biases or biased ‘dark matter’: †* **m** = 15, **M** = 500, **t** = 1e−10 **m** = 15, **M** = 100, **t** = 1e−10	5 ≤ **m** ≤ 100, 5 ≤ **M** ≤ 1000, 1e-03 ≥ **t** ≥ 1e-12
SEG**	*For shorter low-complexity regions (default):* **L** = 12, **K1** = 2.2, **K2** = 2.5 *For longer compositionally biased regions, such as those made of longer tandem repeats:* **L** = 25, **K1** = 3.0, **K2** = 3.3 **L** = 45, **K1** = 3.4, **K2** = 3.75	6 ≤ **L** ≤ 250, 0.2 ≤ **K1** ≤ 4.2, 0.2 ≤ **K2** ≤ 4.2

*The minimum window **m** ≤ maximum window **M**.

**The trigger information entropy threshold **K1** is ≤ **K2** for all runs. The maximum value for **K1** and **K2** in amino-acid sequences is log_2_(20) = 4.3. The recommended parameter sets are taken from the original reference for SEG^5^.

^**†**^Refs.^2,22,23,25,26^.

### Metrics for assessing region discovery

**Figure 2:**
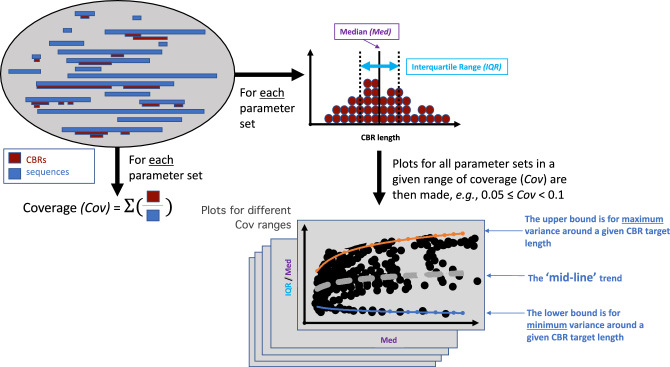
Analyzing the algorithm parameter spaces. A set of protein sequences is analyzed for each parameter set to extract three metrics: (i) the coverage (*Cov*), which is the proportion of the protein data set annotated by the algorithm; (ii) the median length of annotated regions (*Med*); (iii) the interquartile range of the distribution of regions lengths (*IQR*). Plots of *IQR/Med* versus *Med* are derived for intervals of *Cov*, and upper and lower boundary curves are fitted, then average ‘mid-line’ trends calculated. The parameter sets that yield the lower and mid-line bounds are extracted, as described in *Methods*.

To assess and compare parameter set performance, three *metrics* were derived: coverage (*Cov*), median (*Med*), and interquartile range (*IQR*) (Fig. 2).

Coverage (*Cov*) is the proportion of a protein sequence set that is annotated. More liberal parameters lead to greater coverage, and discern more, more mildly-biased regions. *Cov* is calculated taking account of any annotated region overlap. So, residues that appear in multiple annotated regions are counted only once. Median region length (*Med*) and interquartile range (*IQR*) are also calculated from the distribution of region lengths (Fig. 2). *IQR* is an indicator of region length diversity, with smaller *IQR* values for more limited variance in region lengths.

These metrics behave consistently for the two programs and two data sets, Uniref50 and the yeast proteome, with distinct behaviour only for the third data set, the ASTRAL domains, which is to be expected, since it comprises structured regions only (Suppl. Table 1). *IQR/Med* and *Cov* are correlated in all cases, implying that increased coverage comes with an increased range of region sizes. For the UniRef50 data random samples of a third of the size of the sample studied yield highly correlated values for *Cov*, *Med* and *IQR* for both programs (Pearson R^2^ > 0.99), indicating sufficient sample size. The metrics are also highly correlated between the ASTRAL structural domain set and the UniRef50 sample and yeast proteome (Pearson R^2^ > 0.94).

### Deriving curves indicating optimal strategies for CBR/LCR annotation

The behaviour of parameter sets was probed using plots of *IQR/Med* versus *Med*, for intervals of *Cov*. The following *Cov* intervals were used since they have approximately equal numbers of points: 0.015–0.025 (~ 2%), 0.04–0.06 (~ 5%), 0.08–0.12 (~ 12%), 0.2–0.3 (~ 25%), 0.35–0.45 (~ 40%). Upper and lower boundaries for the point distributions were derived for each plot, with logarithmic equations almost universally best fitting (Fig. 2). Boundary points were defined as extreme relative to all the points above or below them within a margin added around the point along the *Med* axis, with different margins (in the range 3–9) being tried, with 3 discovered as optimal. Because of the characteristic banding on these plots, the margin was skewed to lower values for the lower boundary and higher values for the higher (e.g.,* x* − 5 to *x* + 1 for the point *x* for the lower boundary). From the average of these two boundary equations, ‘middle curves’ were calculated (Fig. 2). The points nearest these boundary and middle curves (with a tolerance of ± 0.05 *IQR/Med*) were then analyzed for relationships between metrics and parameters. These relationships were also derived using appropriate line fitting, with characteristic power-law or straight-line relationships between parameters being discovered (discussed in detail in “Results and discussion”). These relationships are robust to missing points that are partitioned to different plots dependent on the *Cov* intervals examined. These were analysed collectively to derive an optimal annotation protocol for LCRs/CBRs of a given target length.

### Further fLPS parameters

There are extra fLPS parameters for: (i) expected amino-acid frequencies (−c option), (ii) initial search granularity (−z option)^15^. The –c option can be: ‘equal’ (= 0.05 for each amino acid), ‘domains’ (frequencies from ASTRAL domains^28^), or ‘user’ (from input sequences). *Cov* shows a clear trend for ‘−c’, with all other parameters set equal: > 99% of the time, ‘user’ yields greatest coverage, then ‘domains’, then ‘equal’. Option –z can be: ‘fast’ (default initial upper P-value = 1e−03), ‘medium’ (= 0.01), and ‘thorough’ (= 0.01). With higher values, biased regions made from longer lists of amino-acid types are detected. Indeed, with other parameters set equal, the mean number of residue types defining CBRs increases from 2.7 (fast) to 3.5 (medium) to 4.3 (thorough), with *Cov* trending similarly (83% of the time thorough > medium > fast).

## Results and discussion

### How do ‘recommended’ parameter sets perform?

**Figure 3:**
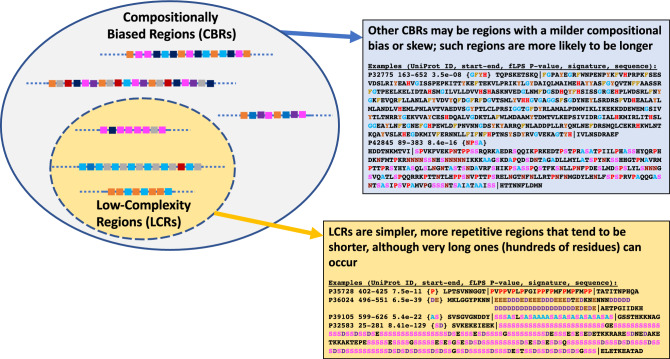
Low-complexity regions (LCRs) can be considered a subset of compositionally biased regions (CBRs). Examples of LCRs and other CBRs are shown that were discovered by fLPS.

What is a low-complexity region (LCR)? What is a compositionally biased region (CBR)? These questions are typically answered by applying the recommended or default parameter sets of programs that annotate them. For example, often LCRs are defined through default application of SEG simply because researchers have always tended to define them that way. However, these default parameters have been quite arbitrarily chosen. Indeed, LCRs and CBRs exist on a spectrum of compositional bias, with LCRs generally shorter and more repetitive, but some cases may also be long (Fig. 3). The most extreme LCRs are, of course, homopeptides^29^.

Also, different parameters can yield widely differing results. Some ‘recommended’ or default parameter sets tend to annotate longer regions, others shorter ones (higher or lower median *(Med)* values in Table 2). Coverage (*Cov*) values vary widely, with default SEG annotating ~ 9% of proteins, but with alternative SEG parameters for longer regions covering > 3 times as many residues. fLPS parameter sets demonstrate a corresponding range of *Med* and *Cov*. Default ‘catch-all’ fLPS parameters yield high coverage (> 60%), since they are designed to comprehensively capture regions with a compositional perturbation; for these parameters, any remaining un-annotated sequence regions can be considered ‘high-complexity’. In general, annotations made by fLPS have a greater diversity of lengths (wider *IQR*), than those made by SEG.

**Table 2:** Metrics for recommended parameters sets for fLPS and SEG.

Program	Type of annotation	Parameters			Metrics								
					*Cov*			*Med*			*IQR/Med*		
		m	M	t	UniRef50	Yeast	ASTRAL	UniRef50	Yeast	ASTRAL	UniRef50	Yeast	ASTRAL
fLPS	Low complexity	5	25	1e−05	0.10	0.07	0.02	25	22	22	1.04	1.05	0.95
	Low complexity	5	25	1e−06	0.08	0.05	0.01	32	27	29	0.94	1.0	0.79
	Default ‘catch-all’	15	500	1e−03	0.68	0.62	0.42	49	43	40	2.43	2.37	1.83
	‘Prion-like’ threshold*	15	100	1e−10	0.19	0.09	0.02	175	122	162	1.05	1.24	0.73
	‘Prion-like’ threshold**	15	500	1e−10	0.35	0.20	0.04	268	195	254	1.32	1.86	0.77

		**L**	**K1**	**K2**	UniRef50	Yeast	ASTRAL	UniRef50	Yeast	ASTRAL	UniRef50	Yeast	ASTRAL
SEG	Default low complexity	12	2.2	2.5	0.09	0.07	0.02	15	15	13	0.47	0.40	0.31
	Longer biased regions***	25	3.0	3.3	0.22	0.17	0.07	39	37	34	0.54	0.49	0.41
	Longer biased regions***	45	3.4	3.75	0.32	0.25	0.11	102	99	88	0.68	0.61	0.49

*Used for identifying prion-like regions or compositionally biased dark matter^2^. For prion-like composition, biases for Q and/or N residues are considered specifically.

**Used for identifying prion-like regions^22,23^, but applied generally to any biases here.

***Parameters for labelling longer CBRs such as those made from tandem repeats with repeat lengths longer than.

The annotations made by Lee et al. are derived from an image-processing algorithm applied to dot plots^17^. These annotations for budding yeast are short with very low length diversity (*Med* = 13; *IQR/Median* = 0.385), and have very low coverage = 2.3%.

Low-complexity or compositionally-biased sequence in structured protein domains is clearly rarer and less diverse lengthwise, regardless of the parameters chosen (Table 1). The ASTRAL set stands out as always having lower *IQR/Med* values, and having much lower *Cov* values generally. Thus, sequence complexity is higher at every resolution in the structured parts of proteins.

### The recommended parameters located in parameter space

**Figure 4:**
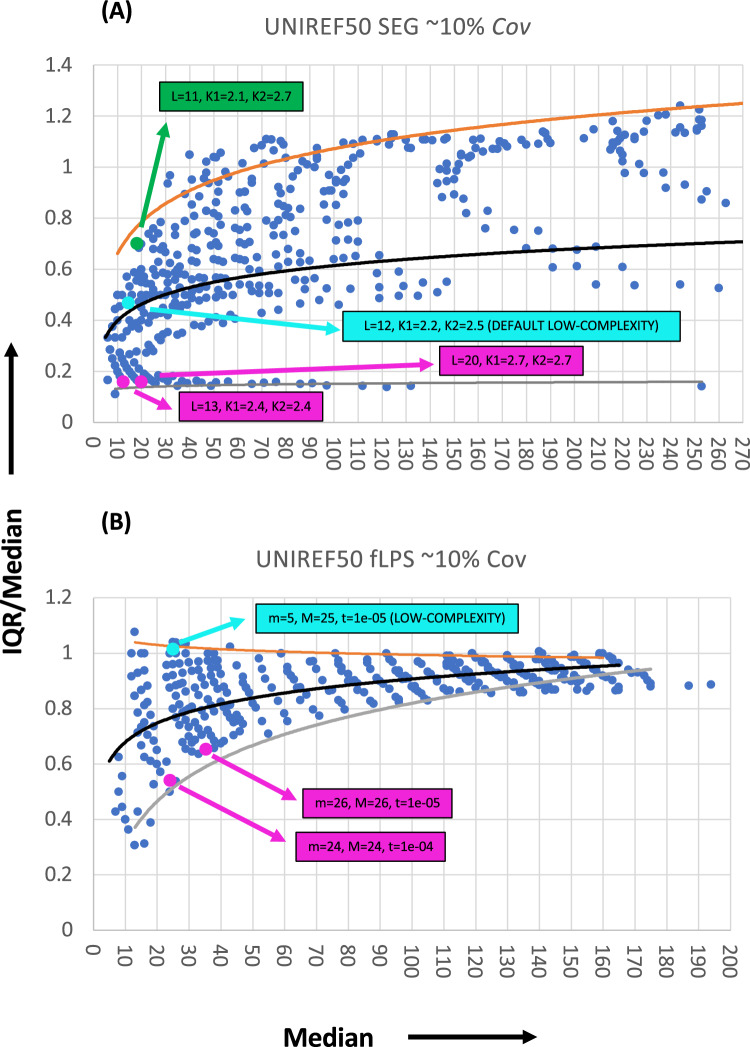
Recommended parameters located within parameter space. Plots of *IQR/Med* versus *Med* for the *Cov* interval 0.08–0.12 (~ 10% coverage) for: (**A**) default SEG parameters, and (**B**) fLPS parameters that have been recommended for annotating shorter, low-complexity regions. The Uniref50 protein sequence data were used. The recommended parameters are labelled in light blue. Examples of parameter sets that are below these and close to the lower boundary are labelled in magenta, while those above and close to the upper boundary are in green.

How do these default or recommended parameter sets compare to the rest of their parameter spaces? How are these parameter sets *special*? To gain answers to these questions, the plots of *IQR/Med* versus *Med* containing the data points for the recommended sets for short LCRs were examined (Fig. 4). Each point in such plots represents a set of parameters. Upper and lower boundaries and mid-lines were calculated as described in *Methods*. The mid-line trend indicates an ’average’ or ‘half-way’ degree of length diversity.

The default SEG parameters are situated very close to the mid-line trend, with a median length of 15 (Fig. 4A). Points near the lower bound can be considered the most narrowly focused around a particular median, and those near the upper bound the most spread out. A typical lower-boundary point just below the default SEG point is highlighted and indicates the typical solution for this extreme of focus, *i.e.*, **K1** trigger entropy threshold = **K2** extension threshold. A similar style of lower-bound solution is indicated for fLPS, with **m** set equal to **M** (Fig. 4B).

The patterning of points on these plots morphs in a distinct way for either algorithm as *Cov* increases (Suppl. Fig. 1). However, at very high *Cov* values (> 0.7 for SEG and > 0.5 for fLPS) they converge to a similar scattering of points and a common limiting behaviour. For such high *Cov*, a lot of proteins are being annotated over their full lengths, and lower *Med* values (and concomitantly higher *IQR* values) simply arise as progressively smaller window lengths pull in annotations of shorter proteins.

### Discovery of trends in program parameters

Depending on their specific proteins of interest and their biological contexts, researchers may have an interest in regions of a particular *target length*, *e.g.,* very short regions that may be motifs, or much longer ones, such as the prion-like regions^22,23,30^. The *Med* value of LCRs/CBRs calculated here can be considered such a target length.

**Figure 5:**
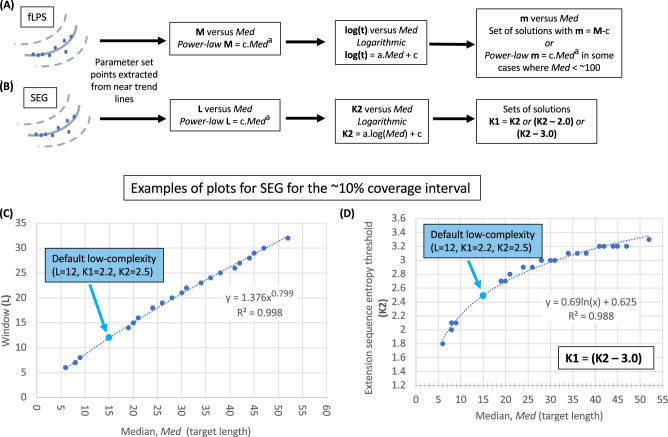
Discovering trends in program parameters. Points near the trendlines in the *IQR/Med* versus *Med* plots are extracted and examined. For both programs (**A**) fLPS and (**B**) SEG, there are power-law relationships between window size and *Med*, and logarithmic relationships with *Med* for a second parameter that measures the degree of bias. Different sets of solutions for the third parameter (**m** for fLPS and **K1** for SEG), then arise in the data. In (**C**) and (**D**), there are examples of plots of *Med* versus **L** and *Med* versus **K2** for a coverage interval of ~ 10% (*Cov* 0.08–0.12).

What program parameters are producing the trend lines in Fig. 4, and how is this related to a specific target length? To answer this, the points near these lines were extracted and the relationship between *Med* and program parameters discerned (as described in *Methods*) (Fig. 5).

For both programs there are standard relationships between this *Med* target length and chosen parameters for the mid-line trends. All correlations for these trends are significant (P < 0.0001). For example, there are power-law relationships between window lengths (**M** for fLPS and **L** for SEG) and *Med* target lengths, with Pearson R^2^ > 0.92 for fLPS and > 0.99 for SEG for all *Cov* ranges studied (Fig. 5A,B). An example of this is shown for SEG and the ~ 10% coverage interval (Fig. 5C). This power-law may thus be an inherent property of such window-based algorithms. Also, logarithmic relationships are standard for the second parameter that determines bias levels (namely the thresholds **K2** for SEG with R^2^ values > 0.95 and **t** for fLPS with R^2^ values > 0.92). An example is shown in Fig. 5D.

The lower-bound trends yield the narrowest focus around a particular target length. The solution of these trends is very simple. For fLPS, **m** = **M** and **M** and **log(t)** are proportional to *Med* and to each other (R^2^ values > 0.89). For SEG, **K1** = **K2,** and **K2** and the window length **L** are proportional to *Med*, and to each other (R^2^ values > 0.92).

However, parameter sets extracted from near the upper bound of these plots for SEG demonstrate that it is not meaningful to consider these solutions. They only exhibit a logarithmic correlation between *Med* and **K2** (*e.g.*, R^2^ = 0.8 for the ~ 10% coverage interval) with both **L** and **K1** being un-correlated with *Med*, and there no subsets with regular patterning of **K1–K2**, as are observed for the other trends (Fig. 5B). This implies that the value of the **K1** trigger threshold is arbitrary, and the annotations thus have no biological meaning, since they are an arbitrary subset of what is possible. This highlights a key feature of the SEG algorithm to be aware of, in that the LCRs/CBRs it annotates are all based on a core region that can have lower sequence entropy than the rest of the LCR. For the fLPS algorithm, these upper bounds are meaningful, with maximum IQR given by **m** set equal to the lowest value 5 and **M** and **t** correlated with target length (*Med*) (*e.g., Med* vs. **M**, R^2^ = 0.98; *Med* vs. **log(t)**, R^2^ = 0.97 for the 10% *Cov* interval). However, in general, for simplicity, we decided not to use upper-bound trends in these analyses.

### Software

**Figure 6:**
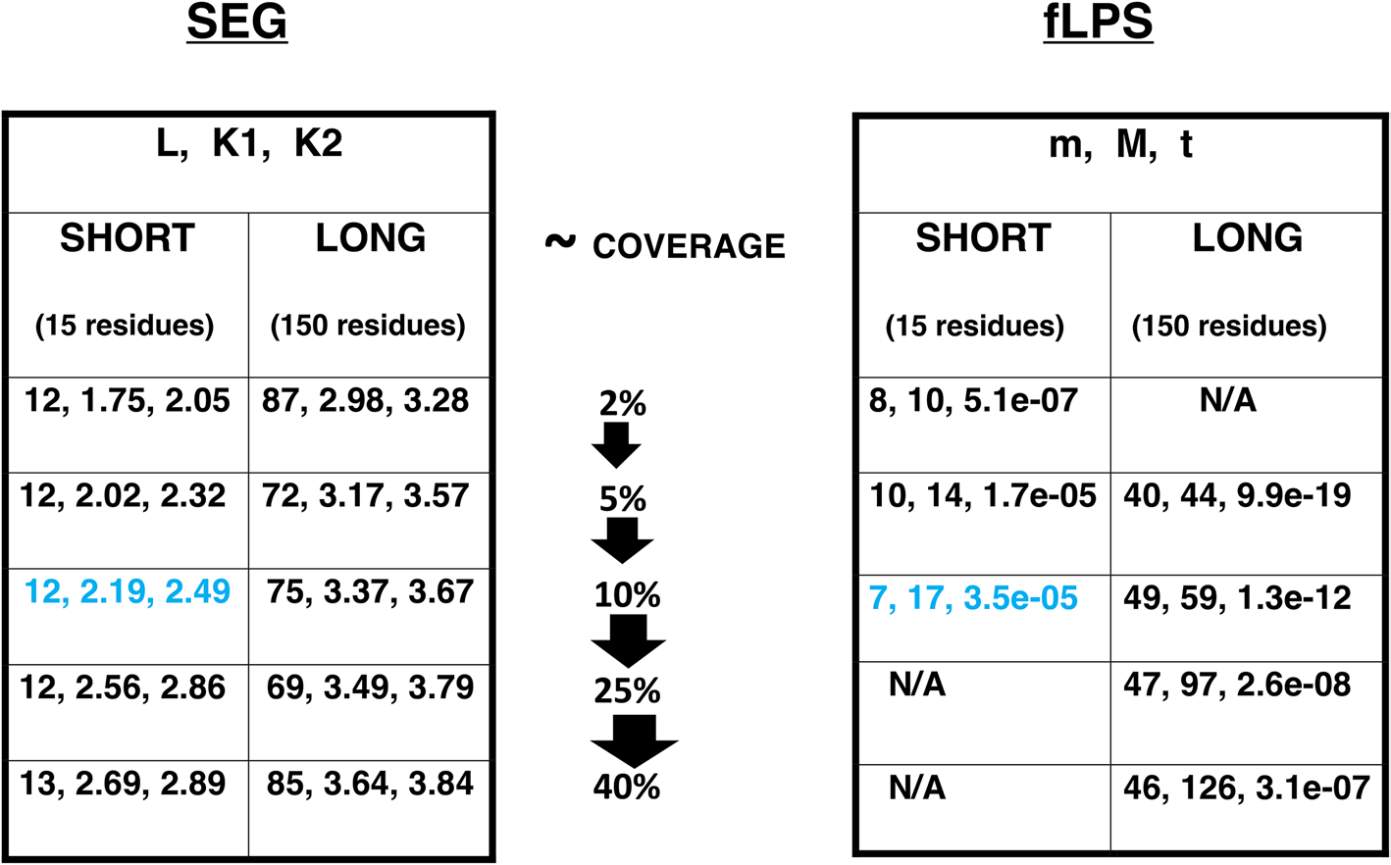
Optimal parameters for a given target length. Considering the median *Med* to be a target length, parameters can be selected to aim at this target. These are listed for a ‘short’ target length (15 residues) and a longer one (150 residues). They are taken from the ‘mid-line’ analysis, which is termed a ‘diverse’ focus in the distributed software *fLPSparameters* and *SEGparameters*. The approximate coverage expected for each parameter set is listed in the middle.

Figure 6 shows how program parameters scale as the implied coverage of annotations increases. Parameters for shorter and longer LCRs/CBRs clearly behave very differently. Also, we can see examples of parameter settings that yield short median lengths and higher coverage, or vice versa*.* The default SEG parameters re-emerge in the table (blue highlights); equivalent fLPS parameters are also highlighted.

A pair of programs **fLPSparameters** and **SEGparameters** to choose parameters to perform such analysis are available at: https://github.com/pmharrison/parameters. They allow for either a ‘diverse’ focus (the mid-line trend) or a ‘narrow’ focus (the lower boundary trend), *i.e.,* with the least possible diversity of region lengths. The discussion below just uses the default ‘diverse’ focus, for greater simplicity.

### An optimized strategy: progressive parsing of CBRs/LCRs annotated across multiple target lengths

So, what is an optimized strategy for annotating regions of a given target length? The best answer is to examine the results from all these progressive program runs (Fig. 6), and assess the biological relevance at each stage. Such an approach may be productive for large-scale bioinformatical analyses involving cross-referencing with other information about function. It may also be useful in directing the parsing of domains to fashion experimental constructs. Thus, what is a meaningfully defined LCR/CBR is determined by such progressive analysis within the relevant biological context for a specific protein under experimental study.

**Figure 7:**
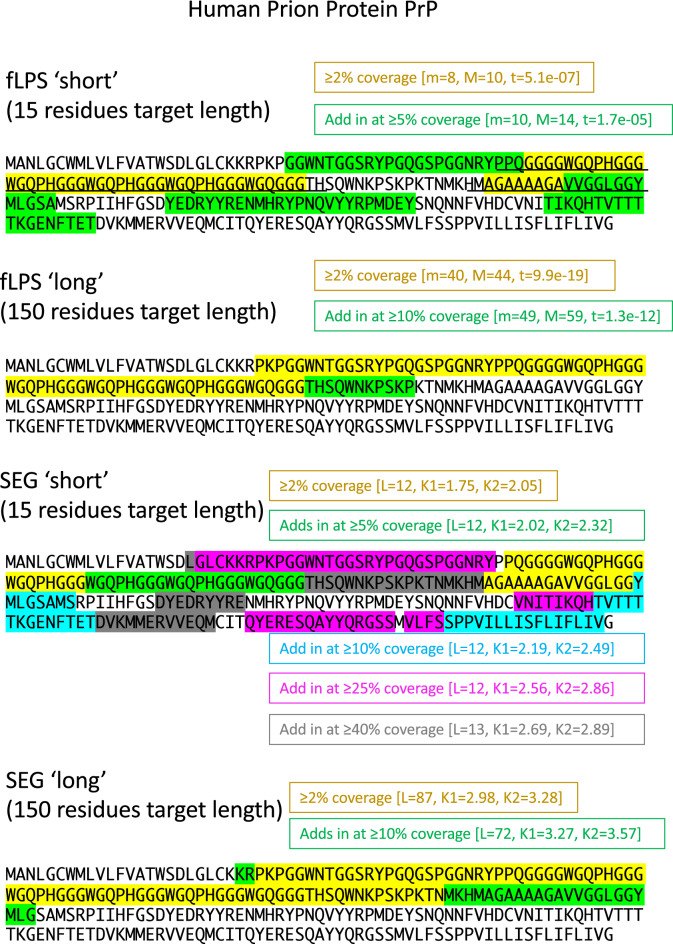
How fLPS and SEG can parse a protein progressively using short and long target lengths. The examples of ‘short’ (15-residue) and ‘long’ (150-residue) target lengths from Fig. 6 are employed on the human prion protein PrP as an example, using a ‘diverse’ focus. The sequence parts that add in at each level are coloured progressively. The definitions of LCRs from Lee et al.^17^ are underlined.

An example of applying these scaled parameter sets is shown (Fig. 7). Human prion protein PrP (UniProt accession P04156) is dissected with parameters for ‘short’ and ‘long’ target lengths (15 and 150 residues). PrP underlies mammalian prion diseases, through amyloid formation, and functions in copper metabolism and circadian control^31^. For fLPS, the ‘long’ parameters annotate the protein’s repetitive copper-binding tract, which converges to a maximum length for parameters with estimated coverage >  ~ 10%. The same tract is also found by ‘long’ SEG parameters, but it is lengthened to include the A/G-rich tract that is transmembrane in some PrP isoforms, and is implicated in conversion to amyloid^32,33^. This region is annotated separately by fLPS for the ‘short’ parameters, along with two other tracts that may be biologically significant. The ‘short’ SEG analysis evidences a slow, gradual filling-in of the whole sequence as estimated coverage rises (Fig. 7).

Further examples of this progressive CBR parsing show that some CBRs are detected regardless of target length, but some are only detected with short target length (Suppl. Fig. 2). For a human collagen (Suppl. Fig. 2B), short proline-rich tracts appear at target length = 15, which then expand into longer regions as estimated coverage is increased. In *Saccharomyces cerevisiae* MRN1 RNA-binding protein (Suppl. Fig. 2C), a putative intrinsically-disordered region up to residue 195 (predicted by AlphaFold, in UniProt entry Q08925) is parsed into distinct sub-regions. Arguably, in general, such results based on parsing CBRs according to compositional biases, rather than sequence entropy, are more meaningful biologically, since sequence entropy per se is less likely to be under selection than, say, a bias for glutamine or glutamate residues linked to specific functional roles.

### Application to searching for CBRs of a given target length

Parameter choice focused on CBR target length can also be used to pick out a data set of CBRs with a similar bias. This is illustrated for the M domain of Sup35 protein from *S. cerevisiae*, a domain which mediates pH sensing during reversible condensate formation in response to stress^34^. A ~ 90-residue {KE}-rich CBR that corresponds to the M-domain was discovered using intermediate target lengths and > 5% estimated coverage (Suppl. Fig. 3A,B); the yeast proteome was then scanned for {KE}/{EK}-rich CBRs of target length = 90 with estimated coverage of 5% (Suppl. Fig. 3C). Significant Gene Ontology category enrichments were observed for these CBRs that are linked to rRNA and ribosomal processing (Suppl. Fig. 3C). Interestingly the Lee et al. annotations do not contain this biased M domain, nor most of the repetitive prion-forming domain (Suppl. Fig. 3A), and only cover ~ 36% of the extent of the {KE}/{EK}-rich regions analyzed here.

### Application to checking parameter dependence of annotations in a large data set

Examining CBR prevalences across a wide range of target lengths can be used to pick out CBR types that are prevalent regardless of parameter choice, and to home in on regions that are only detectable with shorter or longer target lengths, or with lower or higher estimated coverage. This is illustrated for a data set of 137 cancer-associated human intrinsically disordered proteins (IDPs). The top five bias signatures for CBRs are listed for sets of fLPS parameters for target lengths ranging from 10 to 200, and estimated coverage between 2 and 40%.

We can see that some bias signatures are prevalent regardless of parameter choice, e.g., {P}, whereas others are only detected at lower target lengths, *e.g.,* {R} (Suppl. Fig. 4A). {K}-rich regions are only numerous at higher coverage values, *i.e.,* many of them are more mildly-biased. The {R}-rich regions detected are linked to Gene Ontology categories such as protein kinase activity and adenyl nucleotide binding, with {P}-rich regions being associated with enzyme binding and β-catenin binding (Suppl. Fig. 4B). The {R}-rich regions are only detected as prominent, if lower target lengths and medium to high coverage levels are applied.

## Conclusions

An optimized strategy for discovering LCRs/CBRs of a given target length has been derived. Such an approach is suitable for large-scale bioinformatical analyses, for fishing out similar regions of similar length, and in guiding experimental hypotheses about functionally significant protein tracts. We saw how the CBR annotation problem could generally be simplified by choosing specific boundary or mid-line trends. Also, clear highly correlated power-law and logarithmic relationships between target lengths and program parameters were discovered, that were indicated to be general features of window-based algorithms such as fLPS and SEG. This sort of analysis could be combined with application of other tools that can further dissect the character of the CBRs that are discovered, e.g., the LCR server for visualizing repetitiveness^20^, or LCD-Composer, which can assess the dispersion of residue types within a CBR^13^.

The results here are of utility for the development of further improved algorithms for characterization of LCRs and CBRs, and for informing the combination of different algorithms to provide insights into biologically relevant features.

### Supplementary Information

Supplementary Information.

## Data Availability

The data sets analyzed here are available from the public databases UniProt (https://ftp.uniprot.org/pub/databases/uniprot/uniref/uniref50/ and https://www.uniprot.org/proteomes/UP000002311) and SCOPe (http://scop.berkeley.edu/astral/). Results of the research are available in code on GitHub at: https://github.com/pmharrison/parameters.
