# Supplementary material for: Optimizing strategy for the discovery of compositionally-biased or low-complexity regions in proteins
Source: Sci Rep. 2024 Jan 5;14:680. doi: 10.1038/s41598-023-50991-8 (PMC10770407; doi:10.1038/s41598-023-50991-8)
Supplement: Supplementary file 1 — Supplementary Information. [file 41598_2023_50991_MOESM1_ESM.pdf]

Supplementary Material

This material is in the following order:

Suppl. Table 1: Correlations between the metrics.

Suppl. Figure 1: Coverage plots for the parameter spaces of the fLPS and SEG programs.

Suppl. Figure 2: Three additional examples of analysis of CBRs using a scaled progression of parameters for the fLPS program.

Suppl. Figure 3: Analysis of the SUP35 [PSI+]-prion-forming protein, focusing on its {KE}-rich central M-domain, and similar {KE}/{EK}-rich regions.

Suppl. Figure 4: Analysis of CBRs of human cancer-associated IDPs across a wide range of target lengths.

Suppl Table 1: Correlations between the metrics *

	UniRef50			Yeast			ASTRAL		
	Cov	*Med*	*IQR/Med*	*Cov*	*Med*	*IQR/Med*	*Cov*	*Med*	*IQR/Med*
Cov	—	0.06	0.46	—	0.03	0.35	—	–0.04	0.19
Med	0.05	—	0.01	0.01	—	0.08	0.01	—	–0.21
IQR/Med	0.44	0.00	—	0.40	0.08	—	0.24	0.41	—

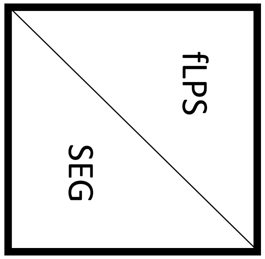

* These are R^2 Pearson correlation coefficients for linear regression. The lower half of each sub-table is for SEG, and the upper half for fLPS, as illustrated by the figure on the right. Equal numbers of parameter sets are used for each data set, for each program separately. P-values are not reported for comparing these correlations, since such results can be made arbitrarily more significant by the inclusion of more data points.

Suppl. Figure 1

Coverage plots for the parameter spaces of the fLPS and SEG programs.

Plots of *IQR/Median* versus Median (*Med*) for intervals of Coverage (*Cov)*

Each point is a set of fLPS or SEG parameters.

(A) The UniRef50 sample of proteins analysed using fLPS for intervals of *Cov* across the UniRef50 sample of proteins.

(B) The UniRef50 sample of proteins analysed using SEG for intervals of *Cov* across the UniRef50 sample of proteins.

(A)

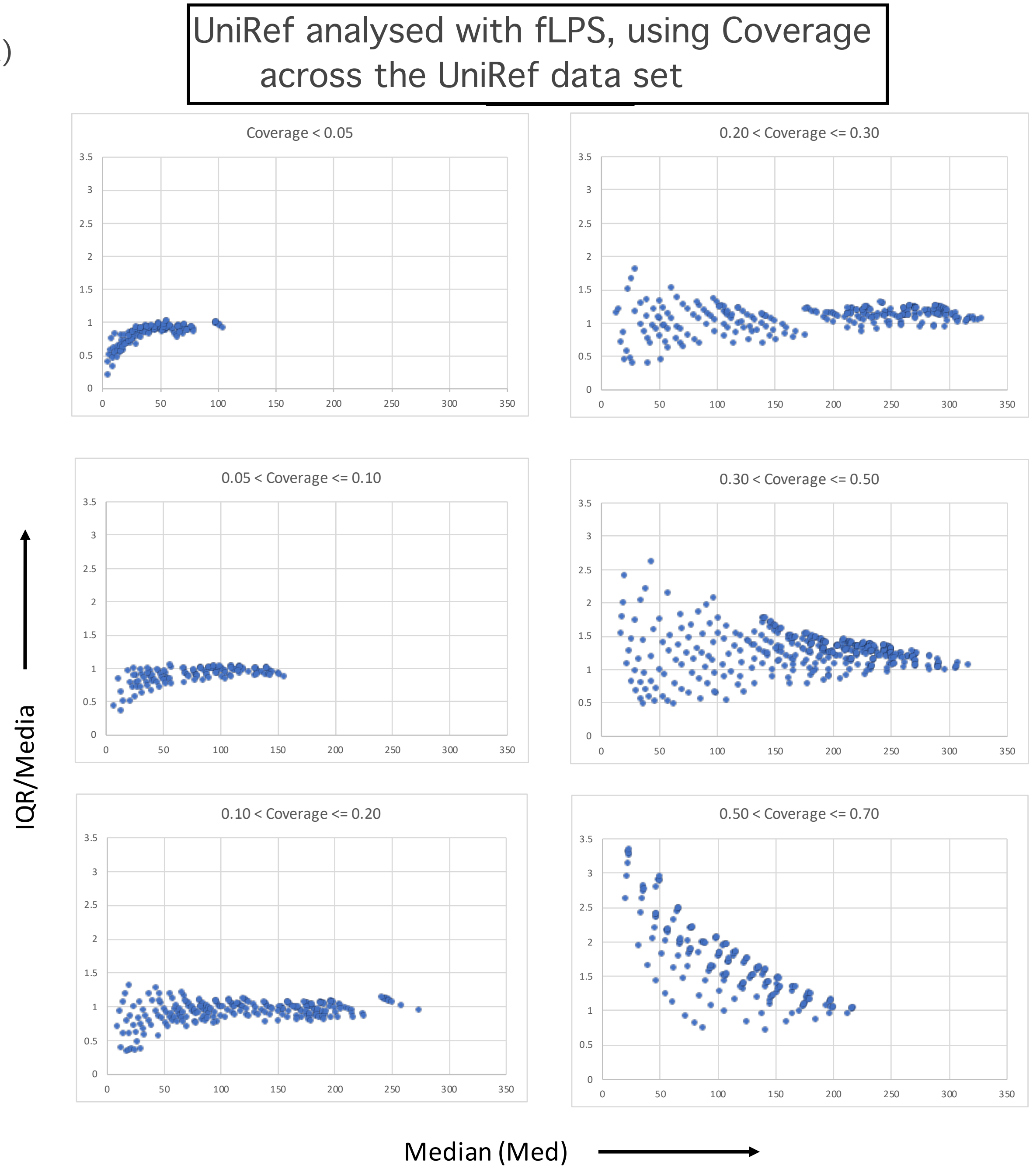

UniRef analysed with fLPS, using Coverage across the UniRef data set
Coverage < 0.05
0.20 < Coverage <= 0.30
0.05 < Coverage <= 0.10
0.30 < Coverage <= 0.50
0.10 < Coverage <= 0.20
0.50 < Coverage <= 0.70
IQR/Media
Median (Med)

(B)

UniRef analysed with SEG, using Coverage across the UniRef data set

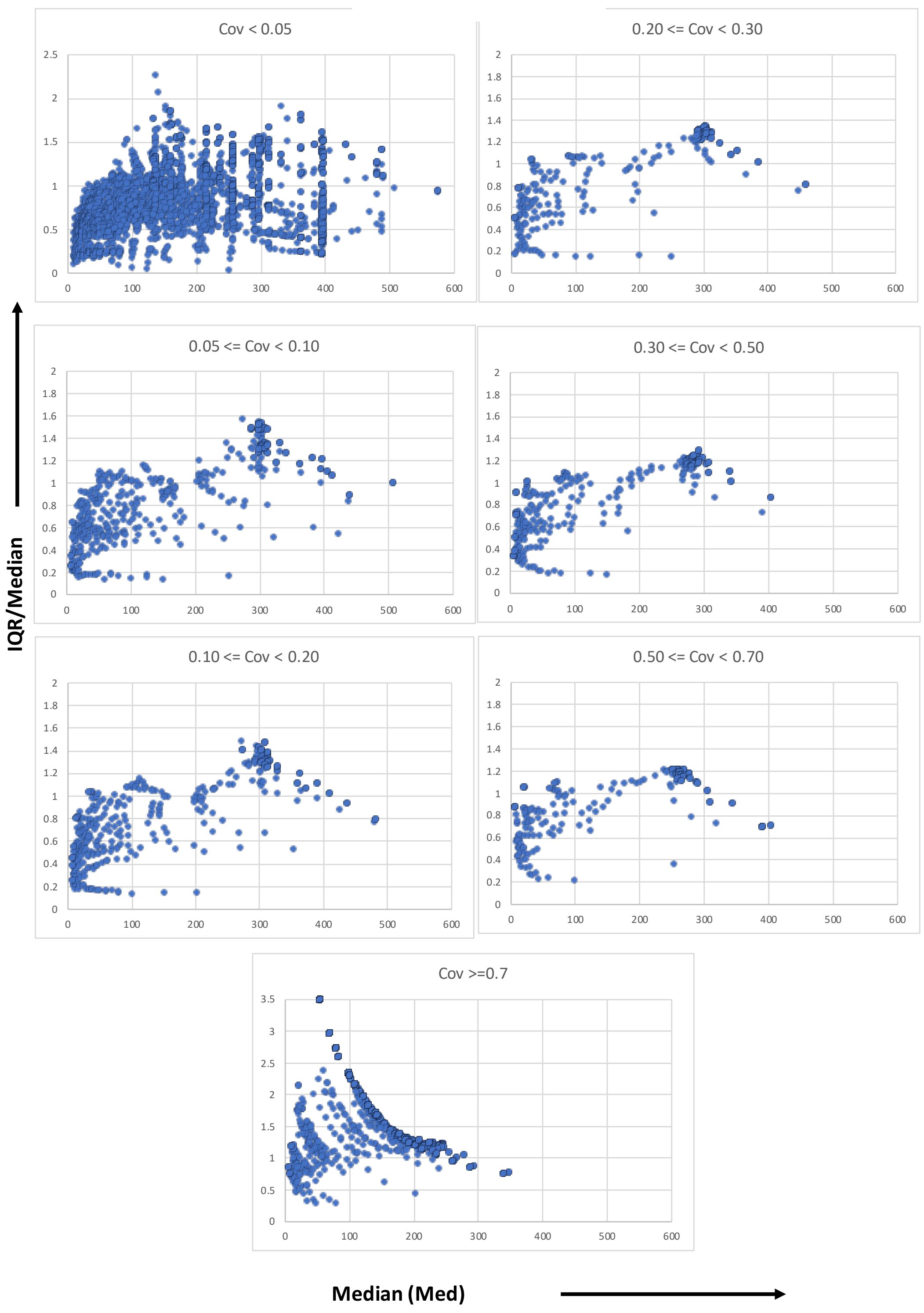

Supplementary Figure 2: Three additional examples of analysis of CBRs using a scaled progression of parameters for the fLPS program

The analysis is performed as for the PrP example in the figure in the main text.

Three target lengths are presented: a ‘short’ one (15 residues), a ‘medium’ one (50 residues) and a ‘long’ one (150 residues).

The key for estimated coverage levels is: yellow (2%), green (5%), cyan (10%), magenta (25%), grey (40%).

The three proteins analyzed are:

(A) P02833 Homeotic protein Antennapedia (D. melanogaster);

(B) Q02388 Collagen alpha-1(VIII) (H. sapiens);

(C) Q08925 RNA-binding protein MRN1 (S. cerevisiae).

Low-complexity regions annotated in the Lee, et al. (2022) paper are single-underlined in the first sequence on each page.

For **(B)**, Q08925 Collagen alpha-1(VIII), a CBR nested within another is double-underlined.

(A) P02833 Homeotic protein Antennapedia (D. melanogaster)

Target length = 15 residues

MTMSTNNCESMTSYFTNSYMGADMHHGHYPGNGVTDLDAQQMHHYSQNANHQGNMPYPRFPPYDRMPYYNGQGMDQQQQHQVYSRPD
SPSSQVGGVMPQAQTNGQLGVPQQQQQQQQQPSQNQQQQQAQQAPQQLQQQLPQVTQQVTHPQQQQQQPVVYASCKLQAAVGGLGMV
PEGGSPPLVDQMSGHHMNAQMTLPHHMGHPQAQLGYTDVGVPDVTEVHQNHHNMGMYQQQSGVPPVGAPPQGMMHQGQGPPQMHQGH
PGQHTPPSQNPNSQSSGMPSPLYPWMRSQFGKCQERKRGRQTYTRYQTLELEKEFHFNRYLTRRRRIEIAHALCLTERQIKIWFQNR
RMKWKKENKTKGEPGSGGEGDEITPPNSPQ

Target length = 50 residues

MTMSTNNCESMTSYFTNSYMGADMHHGHYPGNGVTDLDAQQMHHYSQNANHQGNMPYPRFPPYDRMPYYNGQGMDQQQQHQVYSRPD
SPSSQVGGVMPQAQTNGQLGVPQQQQQQQQQPSQNQQQQQAQQAPQQLQQQLPQVTQQVTHPQQQQQQPVVYASCKLQAAVGGLGMV
PEGGSPPLVDQMSGHHMNAQMTLPHHMGHPQAQLGYTDVGVPDVTEVHQNHHNMGMYQQQSGVPPVGAPPQGMMHQGQGPPQMHQGH
PGQHTPPSQNPNSQSSGMPSPLYPWMRSQFGKCQERKRGRQTYTRYQTLELEKEFHFNRYLTRRRRIEIAHALCLTERQIKIWFQNR
RMKWKKENKTKGEPGSGGEGDEITPPNSPQ

Target length = 150 residues

MTMSTNNCESMTSYFTNSYMGADMHHGHYPGNGVTDLDAQQMHHYSQNANHQGNMPYPRFPPYDRMPYYNGQGMDQQQQHQVYSRPD
SPSSQVGGVMPQAQTNGQLGVPQQQQQQQQQPSQNQQQQQAQQAPQQLQQQLPQVTQQVTHPQQQQQQPVVYASCKLQAAVGGLGMV
PEGGSPPLVDQMSGHHMNAQMTLPHHMGHPQAQLGYTDVGVPDVTEVHQNHHNMGMYQQQSGVPPVGAPPQGMMHQGQGPPQMHQGH
PGQHTPPSQNPNSQSSGMPSPLYPWMRSQFGKCQERKRGRQTYTRYQTLELEKEFHFNRYLTRRRRIEIAHALCLTERQIKIWFQNR
RMKWKKENKTKGEPGSGGEGDEITPPNSPQ

(B) Q02388 Collagen alpha-1(VIII) (H. sapiens)

Target length = 15 residues

MTLRLLVAALCAGILAEAPRVRAQHRERVTCTRLYAADIVFLLDGSSSIGRSNFREVRSFLEGLVLPFSGAASAQGVRFATVQYSDD
PRTEFGLDALGSGGDVIRAIRELSYKGGNTRTGAAILHVADHVFLPQLARPGVPKVCILITDGKSQDLVDTAAQRLKGQGVKLFAVG
IKNADPEELKRVASQPTSDFFFFVNDFSILRTLLPLVSRRVCTTAGGVPVTRPPDDSTSAPRDLVLSEPSSQSLRVQWTAASGPVTG
YKVQYTPLTGLGQPLPSERQEVNVPAGETSVRLRGLRPLTEYQVTVIALYANSIGEAVSGTARTTALEGPELTIQNTTAHSLLVAWR
SVPGATGYRVTWRVLSGGPTQQQELGPGQGSVLLRDLEPGTDYEVTVSTLFGRSVGPATSLMARTDASVEQTLRPVILGPTSILLSW
NLVPEARGYRLEWRRETGLEPPQKVVLPSDVTRYQLDGLQPGTEYRLTLYTLLEGHEVATPATVVPTGPELPVSPVTDLQATELPGQ
RVRVSWSPVPGATQYRIIVRSTQGVERTLVLPGSQTAFDLDDVQAGLSYTVRVSARVGPREGSASVLTVRREPETPLAVPGLRVVVS
DATRVRVAWGPVPGASGFRISWSTGSGPESSQTLPPDSTATDITGLQPGTTYQVAVSVLRGREEGPAAVIVARTDPLGPVRTVHVTQ
ASSSSVTITWTRVPGATGYRVSWHSAHGPEKSQLVSGEATVAELDGLEPDTEYTVHVRAHVAGVDGPPASVVVRTAPEPVGRVSRLQ
ILNASSDVLRITWVGVTGATAYRLAWGRSEGGPMRHQILPGNTDSAEIRGLEGGVSYSVRVTALVGDREGTPVSIVVTTPPEAPPAL
GTLHVVQRGEHSLRLRWEPVPRAQGFLLHWQPEGGQEQSRVLGPELSSYHLDGLEPATQYRVRLSVLGPAGEGPSAEVTARTESPRV
PSIELRVVDTSIDSVTLAWTPVSRASSYILSWRPLRGPGQEVPGSPQTLPGISSSQRVTGLEPGVSYIFSLTPVLDGVRGPEASVTQ
TPVCPRGLADVVFLPHATQDNAHRAEATRRVLERLVLALGPLGPQAVQVGLLSYSHRPSPLFPLNGSHDLGIILQRIRDMPYMDPSG
NNLGTAVVTAHRYMLAPDAPGRRQHVPGVMVLLVDEPLRGDIFSPIREAQASGLNVVMLGMAGADPEQLRRLAPGMDSVQTFFAVDD
GPSLDQAVSGLATALCQASFTTQPRPEPCPVYCPKGQKGEPGEMGLRGQVGPPGDPGLPGRTGAPGPQGPPGSATAKGERGFPGADG
RPGSPGRAGNPGTPGAPGLKGSPGLPGPRGDPGERGPRGPKGEPGAPGQVIGGEGPGLPGRKGDPGPSGPPGPRGPLGDPGPRGPPG
LPGTAMKGDKGDRGERGPPGPGEGGIAPGEPGLPGLPGSPGPQGPVGPPGKKGEKGDSEDGAPGLPGQPGSPGEQGPRGPPGAIGPK
GDRGFPGPLGEAGEKGERGPPGPAGSRGLPGVAGRPGAKGPEGPPGPTGRQGEKGEPGRPGDPAVVGPAVAGPKGEKGDVGPAGPRG
ATGVQGERGPPGLVLPGDPGPKGDPGDRGPIGLTGRAGPPGDSGPPGEKGDPGRPGPPGPVGPRGRDGEVGEKGDEGPPGDPGLPGK
AGERGLRGAPGVRGPVGEKGDQGDPGEDGRNGSPGSSGPKGDRGEPGPPGPPGRLVDTGPGAREKGEPGDRGQEGPRGPKGDPGLPG
APGERGIEGFRGPPGPQGDPGVRGPAGEKGDRGPPGLDGRSGLDGKPGAAGPSGPNGAAGKAGDPGRDGLPGLRGEQGLPGPSGPPG
LPGKPGEDGKPGLNGKNGEPGDPGEDGRKGEKGDSGASGREGRDGPKGERGAPGILGPQGPPGLPGPVGPPGQGFPGVPGGTGPKGD
RGETGSKGEQGLPGERGLRGEPGSVPNVDRLLETAGIKASALREIVETWDESSGSFLPVPERRRGPKGDSGEQGPPGKEGPIGFPGE
RGLKGDRGDPGPQGPPGLALGERGPPGPSGLAGEPGKPGIPGLPGRAGGVGEAGRPGERGERGEKGERGEQGRDGPPGLPGTPGPPG
PPGPKVSVDEPGPGLSGEQGPPGLKGAKGEPGSNGDQGPKGDRGVPGIKGDRGEPGPRGQDGNPGLPGERGMAGPEGKPGLQGPRGP
PGPVGGHGDPGPPGAPGLAGPAGPQGPSGLKGEPGETGPPGRGLTGPTGAVGLPGPPGPSGLVGPQGSPGLPGQVGETGKPGAPGRD
GASGKDGDRGSPGVPGSPGLPGPVGPKGEPGPTGAPGQAVVGLPGAKGEKGAPGGLAGDLVGEPGAKGDRGLPGPRGEKGEAGRAGE
PGDPGEDGQKGAPGPKGFKGDPGVGVPGSPGPPGPPGVKGDLGLPGLPGAPGVVGFPGQTGPRGEMGQPGPSGERGLAGPPGREGIP
GPLGPPGPPGSVGPPGASGLKGDKGDPGVGLPGPRGERGEPGIRGEDGRPGQEGPRGLTGPPGSRGERGEKGDVGSAGLKGDKGDSA
VILGPPGPRGAKGDMGERGPRGLDGDKGPRGDNGDPGDKGSKGEPGDKGSAGLPGLRGLLGPQGQPGAAGIPGDPGSPGKDGVPGIR
GEKGDVGFMGPRGLKGERGVKGACGLDGEKGDKGEAGPPGRPGLAGHKGEMGEPGVPGQSGAPGKEGLIGPKGDRGFDGQPGPKGDQ
GEKGERGTPGIGGFPGPSGNDGSAGPPGPPGSVGPRGPEGLQGQKGERGPPGERVVGAPGVPGAPGERGEQGRPGPAGPRGEKGEAA
LTEDDIRGFVRQEMSQHCACQGQFIASGSRPLPSYAADTAGSQLHAVPVLRVSHAEEEERVPPEDDEYSEYSEYSVEEYQDPEAPWD
SDDPCSLPLDEGSCTAYTLRWYHRAVTGSTEACHPFVYGGCGGNANRFGTREACERRCPPRVVQSQGTG

Target length = 50 residues

MTLRLLVAALCAGILAEAPRVRAQHRERVTCTRLYAADIVFLLDGSSSIGRSNFREVRSFLEGLVLPFSGAASAQGVRFATVQYSDD
PRTEFGLDALGSGGDVIRAIRELSYKGGNTRTGAAILHVADHVFLPQLARPGVPKVCILITDGKSQDLVDTAAQRLKGQGVKLFAVG
IKNADPEELKRVASQPTSDFFFFVNDFSILRTLLPLVSRRVCTTAGGVPVTRPPDDSTSAPRDLVLSEPSSQSLRVQWTAASGPVTG
YKVQYTPLTGLGQPLPSERQEVNVPAGETSVRLRGLRPLTEYQVTVIALYANSIGEAVSGTARTTALEGPELTIQNTTAHSLLVAWR
SVPGATGYRVTWRVLSGGPTQQQELGPGQGSVLLRDLEPGTDYEVTVSTLFGRSVGPATSLMARTDASVEQTLRPVILGPTSILLSW
NLVPEARGYRLEWRRETGLEPPQKVVLPSDVTRYQLDGLQPGTEYRLTLYTLLEGHEVATPATVVPTGPELPVSPVTDLQATELPGQ
RVRVSWSPVPGATQYRIIVRSTQGVERTLVLPGSQTAFDLDDVQAGLSYTVRVSARVGPREGSASVLTVRREPETPLAVPGLRVVVS
DATRVRVAWGPVPGASGFRISWSTGSGPESSQTLPPDSTATDITGLQPGTTYQVAVSVLRGREEGPAAVIVARTDPLGPVRTVHVTQ
ASSSSVTITWTRVPGATGYRVSWHSAHGPEKSQLVSGEATVAELDGLEPDTEYTVHVRAHVAGVDGPPASVVVRTAPEPVGRVSRLQ
ILNASSDVLRITWVGVTGATAYRLAWGRSEGGPMRHQILPGNTDSAEIRGLEGGVSYSVRVTALVGDREGTPVSIVVTTPPEAPPAL
GTLHVVQRGEHSLRLRWEPVPRAQGFLLHWQPEGGQEQSRVLGPELSSYHLDGLEPATQYRVRLSVLGPAGEGPSAEVTARTESPRV
PSIELRVVDTSIDSVTLAWTPVSRASSYILSWRPLRGPGQEVPGSPQTLPGISSSQRVTGLEPGVSYIFSLTPVLDGVRGPEASVTQ

TPVCPRGLADVVFLPHATQDNAHRAEATRRVLERLVLALGPLGPQAVQVGLLSYSHRPSPLFPLNGSHDLGIILQRIRDMPYMDPSG
NNLGTAVVTAHRYMLAPDAPGRRQHVPGVMVLLVDEPLRGDIFSPIREAQASGLNVVMLGMAGADPEQLRRLAPGMDSVQTFFAVDD
GPSLDQAVSGLATALCQASFTTQPRPEPCPVYCPKGQKGEPGEMGLRGQVGPPGDPGLPGRTGAPGPQGPPGSATAKGERGFPGADG
RPGSPGRAGNPGTPGAPGLKGSPGLPGPRGDPGERGPRGPKGEPGAPGQVIGGEGPGLPGRKGDPGPSGPPGPRGPLGDPGPRGPPG
LPGTAMKGDKGDRGERGPPGPGEGGIAPGEPGLPGLPGSPGPQGPVGPPGKKGEKGDSEDGAPGLPGQPGSPGEQGPRGPPGAIGPK
GDRGFPGPLGEAGEKGERGPPGPAGSRGLPGVAGRPGAKGPEGPPGPTGRQGEKGEPGRPGDPAVVGPAVAGPKGEKGDVGPAGPRG
ATGVQGERGPPGLVLPGDPGPKGDPGDRGPIGLTGRAGPPGDSGPPGEKGDPGRPGPPGPVGPRGRDGEVGEKGDEGPPGDPGLPGK
AGERGLRGAPGVRGPVGEKGDQGDPGEDGRNGSPGSSGPKGDRGEPGPPGPPGRLVDTGPGAREKGEPGDRGQEGPRGPKGDPGLPG
APGERGIEGFRGPPGPQGDPGVRGPAGEKGDRGPPGLDGRSGLDGKPGAAGPSGPNGAAGKAGDPGRDGLPGLRGEQGLPGPSGPPG
LPGKPGEDGKPGLNGKNGEPGDPGEDGRKGEKGDSGASGREGRDGPKGERGAPGILGPQGPPGLPGPVGPPGQGFPGVPGGTGPKGD
RGETGSKGEQGLPGERGLRGEPGSVPNVDRLLETAGIKASALREIVETWDESSGSFLPVPERRRGPKGDSGEQGPPGKEGPIGFPGE
RGLKGDRGDPGPQGPPGLALGERGPPGPSGLAGEPGKPGIPGLPGRAGGVGEAGRPGERGERGEKGERGEQGRDGPPGLPGTPGPPG
PPGPKVSVDEPGPGLSGEQGPPGLKGAKGEPGSNGDQGPKGDRGVPGIKGDRGEPGPRGQDGNPGLPGERGMAGPEGKPGLQGPRGP
PGPVGGHGDPGPPGAPGLAGPAGPQGPSGLKGEPGETGPPGRGLTGPTGAVGLPGPPGPSGLVGPQGSPGLPGQVGETGKPGAPGRD
GASGKDGDRGSPGVPGSPGLPGPVGPKGEPGPTGAPGQAVVGLPGAKGEKGAPGGLAGDLVGEPGAKGDRGLPGPRGEKGEAGRAGE
PGDPGEDGQKGAPGPKGFKGDPGVGVPGSPGPPGPPGVKGDLGLPGLPGAPGVVGFPGQTGPRGEMGQPGPSGERGLAGPPGREGIP
GPLGPPGPPGSVGPPGASGLKGDKGDPGVGLPGPRGERGEPGIRGEDGRPGQEGPRGLTGPPGSRGERGEKGDVGSAGLKGDKGDSA
VILGPPGPRGAKGDMGERGPRGLDGDKGPRGDNGDPGDKGSKGEPGDKGSAGLPGLRGLLGPQGQPGAAGIPGDPGSPGKDGVPGIR
GEKGDVGFMGPRGLKGERGVKGACGLDGEKGDKGEAGPPGRPGLAGHKGEMGEPGVPGQSGAPGKEGLIGPKGDRGFDGQPGPKGDQ
GEKGERGTPGIGGFPGPSGNDGSAGPPGPPGSVGPRGPEGLQGQKGERGPPGERVVGAPGVPGAPGERGEQGRPGPAGPRGEKGEAA
LTEDDIRGFVRQEMSQHCACQGQFIASGSRPLPSYAADTAGSQLHAVPVLRVSHAEEEERVPPEDDEYSEYSEYSVEEYQDPEAPWD
SDDPCSLPLDEGSCTAYTLRWYHRAVTGSTEACHPFVYGGCGGNANRFGTREACERRCPPRVVQSQGTG

Target length = 150 residues

MTLRLLVAALCAGILAEAPRVRAQHRERVTCTRLYAADIVFLLDGSSSIGRSNFREVRSFLEGLVLPFSGAASAQGVRFATVQYSDD
PRTEFGLDALGSGGDVIRAIRELSYKGGNTRTGAAILHVADHVFLPQLARPGVPKVCILITDGKSQDLVDTAAQRLKGQGVKLFAVG
IKNADPEELKRVASQPTSDFFFFVNDFSILRTLLPLVSRRVCTTAGGVPVTRPPDDSTSAPRDLVLSEPSSQSLRVQWTAASGPVTG
YKVQYTPLTGLGQPLPSERQEVNVPAGETSVRLRGLRPLTEYQVTVIALYANSIGEAVSGTARTTALEGPELTIQNTTAHSLLVAWR
SVPGATGYRVTWRVLSGGPTQQQELGPGQGSVLLRDLEPGTDYEVTVSTLFGRSVGPATSLMARTDASVEQTLRPVILGPTSILLSW
NLVPEARGYRLEWRRETGLEPPQKVVLPSDVTRYQLDGLQPGTEYRLTLYTLLEGHEVATPATVVPTGPELPVSPVTDLQATELPGQ
RVRVSWSPVPGATQYRIIVRSTQGVERTLVLPGSQTAFDLDDVQAGLSYTVRVSARVGPREGSASVLTVRREPETPLAVPGLRVVVS
DATRVRVAWGPVPGASGFRISWSTGSGPESSQTLPPDSTATDITGLQPGTTYQVAVSVLRGREEGPAAVIVARTDPLGPVRTVHVTQ
ASSSSVTITWTRVPGATGYRVSWHSAHGPEKSQLVSGEATVAELDGLEPDTEYTVHVRAHVAGVDGPPASVVVRTAPEPVGRVSRLQ
ILNASSDVLRITWVGVTGATAYRLAWGRSEGGPMRHQILPGNTDSAEIRGLEGGVSYSVRVTALVGDREGTPVSIVVTTPPEAPPAL
GTLHVVQRGEHSLRLRWEPVPRAQGFLLHWQPEGGQEQSRVLGPELSSYHLDGLEPATQYRVRLSVLGPAGEGPSAEVTARTESPRV
PSIELRVVDTSIDSVTLAWTPVSRASSYILSWRPLRGPGQEVPGSPQTLPGISSSQRVTGLEPGVSYIFSLTPVLDGVRGPEASVTQ
TPVCPRGLADVVFLPHATQDNAHRAEATRRVLERLVLALGPLGPQAVQVGLLSYSHRPSPLFPLNGSHDLGIILQRIRDMPYMDPSG
NNLGTAVVTAHRYMLAPDAPGRRQHVPGVMVLLVDEPLRGDIFSPIREAQASGLNVVMLGMAGADPEQLRRLAPGMDSVQTFFAVDD
GPSLDQAVSGLATALCQASFTTQPRPEPCPVYCPKGQKGEPGEMGLRGQVGPPGDPGLPGRTGAPGPQGPPGSATAKGERGFPGADG
RPGSPGRAGNPGTPGAPGLKGSPGLPGPRGDPGERGPRGPKGEPGAPGQVIGGEGPGLPGRKGDPGPSGPPGPRGPLGDPGPRGPPG
LPGTAMKGDKGDRGERGPPGPGEGGIAPGEPGLPGLPGSPGPQGPVGPPGKKGEKGDSEDGAPGLPGQPGSPGEQGPRGPPGAIGPK
GDRGFPGPLGEAGEKGERGPPGPAGSRGLPGVAGRPGAKGPEGPPGPTGRQGEKGEPGRPGDPAVVGPAVAGPKGEKGDVGPAGPRG
ATGVQGERGPPGLVLPGDPGPKGDPGDRGPIGLTGRAGPPGDSGPPGEKGDPGRPGPPGPVGPRGRDGEVGEKGDEGPPGDPGLPGK
AGERGLRGAPGVRGPVGEKGDQGDPGEDGRNGSPGSSGPKGDRGEPGPPGPPGRLVDTGPGAREKGEPGDRGQEGPRGPKGDPGLPG
APGERGIEGFRGPPGPQGDPGVRGPAGEKGDRGPPGLDGRSGLDGKPGAAGPSGPNGAAGKAGDPGRDGLPGLRGEQGLPGPSGPPG
LPGKPGEDGKPGLNGKNGEPGDPGEDGRKGEKGDSGASGREGRDGPKGERGAPGILGPQGPPGLPGPVGPPGQGFPGVPGGTGPKGD
RGETGSKGEQGLPGERGLRGEPGSVPNVDRLLETAGIKASALREIVETWDESSGSFLPVPERRRGPKGDSGEQGPPGKEGPIGFPGE
RGLKGDRGDPGPQGPPGLALGERGPPGPSGLAGEPGKPGIPGLPGRAGGVGEAGRPGERGERGEKGERGEQGRDGPPGLPGTPGPPG
PPGPKVSVDEPGPGLSGEQGPPGLKGAKGEPGSNGDQGPKGDRGVPGIKGDRGEPGPRGQDGNPGLPGERGMAGPEGKPGLQGPRGP
PGPVGGHGDPGPPGAPGLAGPAGPQGPSGLKGEPGETGPPGRGLTGPTGAVGLPGPPGPSGLVGPQGSPGLPGQVGETGKPGAPGRD
GASGKDGDRGSPGVPGSPGLPGPVGPKGEPGPTGAPGQAVVGLPGAKGEKGAPGGLAGDLVGEPGAKGDRGLPGPRGEKGEAGRAGE
PGDPGEDGQKGAPGPKGFKGDPGVGVPGSPGPPGPPGVKGDLGLPGLPGAPGVVGFPGQTGPRGEMGQPGPSGERGLAGPPGREGIP

GPLGPPGPPGSVGPPGASGLKGDKGDPGVGLPGPRGERGEPGIRGEDGRPGQEGPRGLTGPPGSRGERGEKGDVGSAGLKGDKGDSA
VILGPPGPRGAKGDMGERGPRGLDGDKGPRGDNGDPGDKGSKGEPGDKGSAGLPGLRGLLGPQGQPGAAGIPGDPGSPGKDGVPGIR
GEKGDVGFMGPRGLKGERGVKGACGLDGEKGDKGEAGPPGRPGLAGHKGEMGEPGVPGQSGAPGKEGLIGPKGDRGFDGQPGPKGDQ
GEKGERGTPGIGGFPGPSGNDGSAGPPGPPGSVGPRGPEGLQGQKGERGPPGERVVGAPGVPGAPGERGEQGRPGPAGPRGEKGEAA
LTEDDIRGFVRQEMSQHCACQGQFIASGSRPLPSYAADTAGSQLHAVPVLRVSHAEEEERVPPEDDEYSEYSEYSVEEYQDPEAPWD
SDDPCSLPLDEGSCTAYTLRWYHRAVTGSTEACHPFVYGGCGGNANRFGTREACERRCPPRVVQSQGTG

(C) Q08925 RNA-binding protein MRN1 (S. cerevisiae)

Target length = 15 residues

MVVSYNNNNNNNNNNNNNNNISNNNNNNNMFPPFPSSDDFAMYQQSSSSGPYQETYASGPQNFGDAVYPMNGNFTLLPSDFTREPNDS
FFYENDGIFDYQRIQQQPTQFQTKQRNDSQQQRFSQEQNFEIDNEVVHNNNRYYEYERSSNEVSPFDDENPNVLSDGMSPTIMATAT
AVTNANAPLPVNAQANNPLNFTSAPSRTVYLGNVPPNLSVKELLDHVRSGVVEDVKIIPEKMCAFVSFIDESAALLFHSDAILKRLN
IGDRDIKIGWGKPTRIDPIVAARISTDGATRNVYIGRMTIEGEESHLSEEQLRVDLEEYGEIDCIKIIKEKGIAFIHFASILNAIKV
VTNLPIRNPYYQNKRIFYGKDRCAFITKTQQHNAAQFLGVQPGMEHMIEFSDREFISNALLQQSAAAAAIATSAGGPNNLGNRTVYL
GSLPKDVKIEEICNAVRGGLLQSIKLLNDRYVCFVTFIDPTAAAQFYAMSSLYGFTVQKKRCKVGWGKHSGPLPNALALAVSNGASR
NVYVGNIDFVGDSLRDERVFTESNLRHIFQQYGEVEQINFLPEKNCCFINYTNISNAILALDKIKSNPYFKDLKINFGKDRCGNVPH
QSR

Target length = 50 residues

MVVSYNNNNNNNNNNNNNNNISNNNNNNNMFPPFPSSDDFAMYQQSSSSGPYQETYASGPQNFGDAVYPMNGNFTLLPSDFTREPNDS
FFYENDGIFDYQRIQQQPTQFQTKQRNDSQQQRFSQEQNFEIDNEVVHNNNRYYEYERSSNEVSPFDDENPNVLSDGMSPTIMATAT
AVTNANAPLPVNAQANNPLNFTSAPSRTVYLGNVPPNLSVKELLDHVRSGVVEDVKIIPEKMCAFVSFIDESAALLFHSDAILKRLN
IGDRDIKIGWGKPTRIDPIVAARISTDGATRNVYIGRMTIEGEESHLSEEQLRVDLEEYGEIDCIKIIKEKGIAFIHFASILNAIKV
VTNLPIRNPYYQNKRIFYGKDRCAFITKTQQHNAAQFLGVQPGMEHMIEFSDREFISNALLQQSAAAAAIATSAGGPNNLGNRTVYL
GSLPKDVKIEEICNAVRGGLLQSIKLLNDRYVCFVTFIDPTAAAQFYAMSSLYGFTVQKKRCKVGWGKHSGPLPNALALAVSNGASR
NVYVGNIDFVGDSLRDERVFTESNLRHIFQQYGEVEQINFLPEKNCCFINYTNISNAILALDKIKSNPYFKDLKINFGKDRCGNVPH
QSR

Target length = 150 residues

MVVSYNNNNNNNNNNNNNNNISNNNNNNNMFPPFPSSDDFAMYQQSSSSGPYQETYASGPQNFGDAVYPMNGNFTLLPSDFTREPNDS
FFYENDGIFDYQRIQQQPTQFQTKQRNDSQQQRFSQEQNFEIDNEVVHNNNRYYEYERSSNEVSPFDDENPNVLSDGMSPTIMATAT
AVTNANAPLPVNAQANNPLNFTSAPSRTVYLGNVPPNLSVKELLDHVRSGVVEDVKIIPEKMCAFVSFIDESAALLFHSDAILKRLN
IGDRDIKIGWGKPTRIDPIVAARISTDGATRNVYIGRMTIEGEESHLSEEQLRVDLEEYGEIDCIKIIKEKGIAFIHFASILNAIKV
VTNLPIRNPYYQNKRIFYGKDRCAFITKTQQHNAAQFLGVQPGMEHMIEFSDREFISNALLQQSAAAAAIATSAGGPNNLGNRTVYL
GSLPKDVKIEEICNAVRGGLLQSIKLLNDRYVCFVTFIDPTAAAQFYAMSSLYGFTVQKKRCKVGWGKHSGPLPNALALAVSNGASR
NVYVGNIDFVGDSLRDERVFTESNLRHIFQQYGEVEQINFLPEKNCCFINYTNISNAILALDKIKSNPYFKDLKINFGKDRCGNVPH
QSR

Supplementary Figure 3: Analysis of the SUP35 [PSI+]-prion-forming protein, focusing on its {KE}-rich central M-domain, and similar {KE}/{EK}-rich regions

In part **(A)**, the analysis is performed as for the PrP example in the figure in the main text. Three target lengths are presented: a 'short' one (15 residues), a 'medium' one (50 residues) and a 'long' one (150 residues). The key for estimated coverage levels is: yellow (2%), green (5%), cyan (10%), magenta (25%), grey (40%). The boundary residues are labelled in red for the prion-forming domain, the N-terminal prion-nucleation domain within it, and the charged M domain. The {KE}-rich domain approximately corresponding to the M domain is double-underlined. This region is first found at the 5% coverage level. Low-complexity regions annotated in the Lee, et al. (2022) paper are single-underlined in the first sequence on each page.

(B) Same as in **(A)**, but except for the SEG algorithm. Double-underlined is the low-complexity domain that most closely corresponds with the Sup35 charged M domain, found with target length = 50 residues, and coverage level 10%.

(C) Gene ontology category enrichments for {KE}/{EK}-rich domains found in the *S. cerevisiae* proteome, using fLPS parameters for target length = 90 residues and estimated coverage of 5%. These are calculated using the Gorilla Gene Ontology tool https://cbl-gorilla.cs.technion.ac.il/GOrilla .

(A) Analysis of SUP35 sequence using three target lengths and the fLPS program

End of N-terminal prion nucleation

End of prion-forming domain, Start of charged M domain

End of charged M domain

Target length = 15 residues

MSDSNQGNNQQNYQQYSQNGNQQQGNNRYQGYQAYNAQAQPAGGYYQNYQGYSGYQQGGYQQYNPDAGYQQQYNPQGGYQQYNPQGG
YQQQFNPQGGRGNYKNFNYNNNLQGYQAGFQPQSQGMSLNDFQKQQKQAAPKPKKTLKLVSSSGIKLANATKKVGTKPAESDKKEEE
KSAETKEPTKEPTKVEEPVKKEEKPVQTEEKTEEKSELPKVEDLKISESTHNTNNANVTSADALIKEQEEEVDDEVVNDMFGGKDHV
SLIFMGHVDAGKSTMGGNLLYLTGSVDKRTIEKYEREAKDAGRQGWYLSWVMDTNKEERNDGKTIEVGKAYFETEKRRYTILDAPGH
KMYVSEMIGGASQADVGVLVISARKGEYETGFERGGQTREHALLAKTQGVNKMVVVVNKMDDPTVNWSKERYDQCVSNVSNFLRAIG
YNIKTDVVFMPVSGYSGANLKDHVDPKECPWYTGPTLLEYLDTMNHVDRHINAPFMLPIAAKMKDLGTIVEGKIESGHIKKGQSTLL
MPNKTAVEIQNIYNETENEVDMAMCGEQVKLRIKGVEEEDISPGFVLTSPKNPIKSVTKFVAQIAIVELKSIIAAGFSCVMHVHTAI
EEVHIVKLLHKLEKGTNRKSKKPPAFAKKGMKVIAVLETEAPVCVETYQDYPQLGRFTLRDQGTTIAIGKIVKIAE

Target length = 50 residues

MSDSNQGNNQQNYQQYSQNGNQQQGNNRYQGYQAYNAQAQPAGGYYQNYQGYSGYQQGGYQQYNPDAGYQQQYNPQGGYQQYNPQGG
YQQQFNPQGGRGNYKNFNYNNNLQGYQAGFQPQSQGMSLNDFQKQQKQAAPKPKKTLKLVSSSGIKLANATKKVGTKPAESDKKEEE
KSAETKEPTKEPTKVEEPVKKEEKPVQTEEKTEEKSELPKVEDLKISESTHNTNNANVTSADALIKEQEEEVDDEVVNDMFGGKDHV
SLIFMGHVDAGKSTMGGNLLYLTGSVDKRTIEKYEREAKDAGRQGWYLSWVMDTNKEERNDGKTIEVGKAYFETEKRRYTILDAPGH
KMYVSEMIGGASQADVGVLVISARKGEYETGFERGGQTREHALLAKTQGVNKMVVVVNKMDDPTVNWSKERYDQCVSNVSNFLRAIG
YNIKTDVVFMPVSGYSGANLKDHVDPKECPWYTGPTLLEYLDTMNHVDRHINAPFMLPIAAKMKDLGTIVEGKIESGHIKKGQSTLL
MPNKTAVEIQNIYNETENEVDMAMCGEQVKLRIKGVEEEDISPGFVLTSPKNPIKSVTKFVAQIAIVELKSIIAAGFSCVMHVHTAI
EEVHIVKLLHKLEKGTNRKSKKPPAFAKKGMKVIAVLETEAPVCVETYQDYPQLGRFTLRDQGTTIAIGKIVKIAE

Target length = 150 residues

MSDSNQGNNQQNYQQYSQNGNQQQGNNRYQGYQAYNAQAQPAGGYYQNYQGYSGYQQGGYQQYNPDAGYQQQYNPQGGYQQYNPQGG
YQQQFNPQGGRGNYKNFNYNNNLQGYQAGFQPQSQGMSLNDFQKQQKQAAPKPKKTLKLVSSSGIKLANATKKVGTKPAESDKKEEE
KSAETKEPTKEPTKVEEPVKKEEKPVQTEEKTEEKSELPKVEDLKISESTHNTNNANVTSADALIKEQEEEVDDEVVNDMFGGKDHV
SLIFMGHVDAGKSTMGGNLLYLTGSVDKRTIEKYEREAKDAGRQGWYLSWVMDTNKEERNDGKTIEVGKAYFETEKRRYTILDAPGH
KMYVSEMIGGASQADVGVLVISARKGEYETGFERGGQTREHALLAKTQGVNKMVVVVNKMDDPTVNWSKERYDQCVSNVSNFLRAIG
YNIKTDVVFMPVSGYSGANLKDHVDPKECPWYTGPTLLEYLDTMNHVDRHINAPFMLPIAAKMKDLGTIVEGKIESGHIKKGQSTLL
MPNKTAVEIQNIYNETENEVDMAMCGEQVKLRIKGVEEEDISPGFVLTSPKNPIKSVTKFVAQIAIVELKSIIAAGFSCVMHVHTAI
EEVHIVKLLHKLEKGTNRKSKKPPAFAKKGMKVIAVLETEAPVCVETYQDYPQLGRFTLRDQGTTIAIGKIVKIAE

(B) Analysis of SUP35 sequence using three target lengths and the SEG program

End of N-terminal prion nucleation

End of prion-forming domain, Start of charged M domain

End of charged M domain

Target length = 15 residues

```
MSDSNQGNNQQNYQQYSQNGNQQQGNNRYQGYQAYNAQAQPAGGYYQNYQGYSGYQQGGYQQYNPDAGYQQQYNPQGG
YQQQFNPQGGRGNYKNFNYNNNLQGYQAGFQPQSQGMSLNDFQKQQKQAAPKPKKTLKLVSSSGIKLANATKKVGTKPAESDKKEEE
KSAETKEPTKEPTKVEEPVKKEEKPVQTEEKTEEKSELPKVEDLKISESTHNTNNANVTSADALIKEQEEEVDDEVVNDMFGGKDHV
SLIFMGHVDAGKSTMGGNLLYLTGSVDKRTIEKYEREAKDAGRQGWYLSWVMDTNKEERNDGKTIEVGKAYFETEKRRYTILDAPGH
KMYVSEMIGGASQADVGVLVISARKGEYETGFERGGQTREHALLAKTQGVNKMVVVVNKMDDPTVNWSKERYDQCVSNVSNFLRAIG
YNIKTDVVFMPVSGYSGANLKDHVDPKECPWYTGPTLLEYLDTMNHVDRHINAPFMLPIAAKMKDLGTIVEGKIESGHIKKGQSTLL
MPNKTAVEIQNIYNETENEVDMAMCGEQVKLRIKGVEEEDISPGFVLTSPKNPIKSVTKFVAQIAIVELKSIIAAGFSCVMHVHTAI
EEVHIVKLLHKLEKGTNRKSKKPPAFAKKGMKVIAVLETEAPVCVETYQDYPQLGRFTLRDQGTTIAIGKIVKIAE
.........1.........2.........3.........4.........5.........6.........7.........8
123456789.123456789.123456789.123456789
```

Target length = 50 residues

```
MSDSNQGNNQQNYQQYSQNGNQQQGNNRYQGYQAYNAQAQPAGGYYQNYQGYSGYQQGGYQQYNPDAGYQQQYNPQGGYQQYNPQGG
YQQQFNPQGGRGNYKNFNYNNNLQGYQAGFQPQSQGMSLNDFQKQQKQAAPKPKKTLKLVSSSGIKLANATKKVGTKPAESDKKEEE
KSAETKEPTKEPTKVEEPVKKEEKPVQTEEKTEEKSELPKVEDLKISESTHNTNNANVTSADALIKEQEEEVDDEVVNDMFGGKDHV
SLIFMGHVDAGKSTMGGNLLYLTGSVDKRTIEKYEREAKDAGRQGWYLSWVMDTNKEERNDGKTIEVGKAYFETEKRRYTILDAPGH
KMYVSEMIGGASQADVGVLVISARKGEYETGFERGGQTREHALLAKTQGVNKMVVVVNKMDDPTVNWSKERYDQCVSNVSNFLRAIG
YNIKTDVVFMPVSGYSGANLKDHVDPKECPWYTGPTLLEYLDTMNHVDRHINAPFMLPIAAKMKDLGTIVEGKIESGHIKKGQSTLL
MPNKTAVEIQNIYNETENEVDMAMCGEQVKLRIKGVEEEDISPGFVLTSPKNPIKSVTKFVAQIAIVELKSIIAAGFSCVMHVHTAI
EEVHIVKLLHKLEKGTNRKSKKPPAFAKKGMKVIAVLETEAPVCVETYQDYPQLGRFTLRDQGTTIAIGKIVKIAE
```

Target length = 150 residues

```
MSDSNQGNNQQNYQQYSQNGNQQQGNNRYQGYQAYNAQAQPAGGYYQNYQGYSGYQQGGYQQYNPDAGYQQQYNPQGGYQQYNPQGG
YQQQFNPQGGRGNYKNFNYNNNLQGYQAGFQPQSQGMSLNDFQKQQKQAAPKPKKTLKLVSSSGIKLANATKKVGTKPAESDKKEEE
KSAETKEPTKEPTKVEEPVKKEEKPVQTEEKTEEKSELPKVEDLKISESTHNTNNANVTSADALIKEQEEEVDDEVVNDMFGGKDHV
SLIFMGHVDAGKSTMGGNLLYLTGSVDKRTIEKYEREAKDAGRQGWYLSWVMDTNKEERNDGKTIEVGKAYFETEKRRYTILDAPGH
KMYVSEMIGGASQADVGVLVISARKGEYETGFERGGQTREHALLAKTQGVNKMVVVVNKMDDPTVNWSKERYDQCVSNVSNFLRAIG
YNIKTDVVFMPVSGYSGANLKDHVDPKECPWYTGPTLLEYLDTMNHVDRHINAPFMLPIAAKMKDLGTIVEGKIESGHIKKGQSTLL
MPNKTAVEIQNIYNETENEVDMAMCGEQVKLRIKGVEEEDISPGFVLTSPKNPIKSVTKFVAQIAIVELKSIIAAGFSCVMHVHTAI
EEVHIVKLLHKLEKGTNRKSKKPPAFAKKGMKVIAVLETEAPVCVETYQDYPQLGRFTLRDQGTTIAIGKIVKIAE
```

(C) Enrichments of GO terms for {KE}/{EK}-rich domains found in the S. cerevisiae proteome, using parameters for target length = 90 residues and estimated coverage of 5%

Gene Ontology term	Term Description	Corrected P-value	Number with term (Total proteins=29)
PROCESS			
0016072	rRNA metabolic process	8.2e-03	10
0071840	Cellular component organization or biogenesis	5.3e-03	23
0042254	Ribosome biogenesis	7.6e-03	8
0022613	Ribonucleoprotein complex biogenesis	1.5e-02	8
0044085	Cellular component biogenesis	2.6e-02	8
0006364	rRNA processing	3.2e-02	8
COMPONENT			
0005730	nucleolus	6.5e-04	10
0030686	90S preribosome	1.6e-02	5
0043232	Intracellular Non-membrane-bound organelle	1.0e-02	15
0005654	Nucleoplasm	2.4e-02	4
0030684	Preribosome	2.1e-02	6
0048471	Perinuclear region of cytoplasm	2.2e-02	3
0032991	Protein-containing complex	2.7e-02	21
0044428	Nuclear part	2.5e-02	15

Suppl. Figure 4: Analysis of CBRs of human cancer-associated IDPs across a wide range of target lengths

(A) Top five CBR biases for a wide range of target lengths and estimated coverages. fLPS is run using default ‘domains’ background frequencies. The default ‘diverse’ focus of fLPSparameters was used. Biases for the signatures {P}, {E}, {K}, {R}, {Q} and {PQ} are highlighted. Parameter combinations that are not calculable are labelled ‘n/a’.

(B) Examples of enrichments for Gene Ontology Function categories. Up to ten top-ranking enrichments are shown for each example. These enrichments are calculated with the Gorilla tool (https://cbl-gorilla.cs.technion.ac.il/GOrilla/). The examples from the table are (bias signature, target length, estimated coverage): {R}, 15, 10%; {R}, 25, 40%; {P}, 10, 5%; {P}, 25, 10%. GO categories shared between the two {R} examples or between the two {P} examples are in **bold**, otherwise in *italics*.

(A) Analysis of prevalent biases in human cancer-associated IDPs across a range of target lengths

Coverage→ (rows: Target Length)	2%		5%		10%		25%		40%	
10	{S}	26	{P}	60	n/a		n/a		n/a	
	{P}	25	{S}	55						
	{E}	14	{E}	33						
	{A}	12	{R}	27						
	{G}	10	{Q}	24						
15	{P}	28	{S}	49	{P}	66	n/a		n/a	
	{S}	19	{P}	47	{S}	62				
	{E}	9	{E}	20	{E}	34				
	{A}	9	{K}	19	{K}	28				
	{Q}	6	{A}	18	{R}	27				
25	{P}	23	{P}	42	{P}	53	{S}	97	{S}	104
	{S}	15	{S}	34	{S}	51	{P}	91	{P}	100
	{Q}	5	{E}	15	{E}	23	{Q}	64	{Q}	71
	{E}	5	{C}	11	{K}	18	{E}	61	{R}	68
	{PQ}	4	{A}	11	{Q}	17	{K}	59	{E}	68
40	{P}	14	{P}	28	{P}	46	{S}	83	{S}	95
	{S}	7	{S}	21	{S}	43	{P}	83	{P}	91
	{E}	4	{G}	9	{C}	17	{E}	50	{Q}	61
	{PQ}	3	{C}	9	{E}	15	{K}	40	{E}	57
	{G}	3	{Q}	7	{G}	14	{C}	39	{K}	54
70	{P}	8	{P}	18	{P}	45	{P}	88	{S}	102
	{S}	4	{S}	11	{S}	27	{S}	66	{P}	89
	{SQ}	2	{Q}	6	{C}	13	{C}	31	{E}	43
	{Q}	2	{C}	5	{Q}	11	{Q}	20	{C}	43
	{PQ}	2	{PS}	5	{PS}	9	{K}	20	{K}	31
100	{P}	8	{P}	13	{P}	32	{P}	53	{S}	93
	{S}	2	{S}	7	{S}	19	{S}	49	{P}	76
	{Q}	2	{Q}	4	{G}	10	{C}	17	{C}	36
	{PQ}	2	{C}	4	{C}	7	{E}	15	{E}	25
	{PH}	2	{STH}	2	{Q}	7	{Q}	12	{K}	25
150	n/a		{P}	9	{P}	17	{S}	49	{P}	55
			{S}	4	{S}	12	{P}	42	{S}	54
			{Q}	3	{C}	6	{C}	13	{C}	18
			{PG}	3	{Q}	5	{G}	12	{Q}	13

Target Length →

		{PS} 2	{SP} 4	{E} 10	{E} 13
**200**	n/a	{P} 8	{P} 13	{S} 32	{P} 42
		{S} 3	{S} 9	{P} 31	{S} 41
		{Q} 3	{PS} 4	{G} 10	{C} 12
		{T} 1	{C} 4	{Q} 8	{G} 11
		{TSP} 1	{SQ} 3	{C} 7	{Q} 10

(B) Gene Ontology Function category enrichments for examples

{R} , 15, 10%

GO term	Description	Corrected P-value	Count
GO:0140097	*catalytic activity, acting on DNA*	*4.88E-2*	*4/27*
**GO:0032559**	**adenyl ribonucleotide binding**	**4.79E-2**	**9/27**
**GO:0030554**	**adenyl nucleotide binding**	**4.84E-2**	**9/27**

{R} , 25, 40%

GO term	Description	Corrected P-value	Count
GO:0004714	*transmembrane receptor protein tyrosine kinase activity*	*2.1E-7*	*8/67*
GO:0019199	*transmembrane receptor protein kinase activity*	*9.81E-7*	*8/67*
GO:0005524	*ATP binding*	*5.03E-6*	*22/67*
GO:0016772	*transferase activity, transferring phosphorus-containing groups*	*5.15E-6*	*17/67*
GO:0004672	*protein kinase activity*	*4.66E-6*	*14/67*
**GO:0032559**	**adenyl ribonucleotide binding**	**5.36E-6**	**22/67**

**GO:0030554**	**adenyl nucleotide binding**	**5.37E-6**	**22/67**
GO:0000166	*nucleotide binding*	*1.51E-5*	*25/67*
GO:1901265	*nucleoside phosphate binding*	*1.36E-5*	*25/67*
GO:0004713	*protein tyrosine kinase activity*	*1.28E-5*	*8/67*

{P}, 10, 5%

GO term	Description	Corrected P-value	Count
**GO:0019899**	**enzyme binding**	**1.15E-4**	**23/60**
GO:0008134	*transcription factor binding*	*1.19E-4*	*13/60*
**GO:0008013**	**beta-catenin binding**	**4.29E-4**	**6/60**
**GO:0001085**	**RNA polymerase II transcription factor binding**	**3.8E-4**	**7/60**
**GO:0003677**	**DNA binding**	**3.8E-4**	**23/60**
**GO:0004672**	**protein kinase activity**	**5.9E-4**	**11/60**
**GO:1901363**	**heterocyclic compound binding**	**1.02E-3**	**36/60**
**GO:0016772**	**transferase activity, transferring phosphorus-containing groups**	**9.81E-4**	**13/60**
GO:0008022	*protein C-terminus binding*	*9.22E-4*	*7/60*
**GO:0097159**	**organic cyclic compound binding**	**1.02E-3**	**36/60**

{P}, 25, 10%

GO term	Description	Corrected P-value	Count
**GO:0019899**	**enzyme binding**	**9.64E-6**	**23/53**
GO:0019900	*kinase binding*	*2.37E-4*	*13/53*

GO:0004713	*protein tyrosine kinase activity*	*1.6E-4*	*7/53*
**GO:0008013**	**beta-catenin binding**	**1.68E-4**	**6/53**
**GO:0001085**	**RNA polymerase II transcription factor binding**	**1.43E-4**	**7/53**
**GO:0004672**	**protein kinase activity**	**1.88E-4**	**11/53**
**GO:0016772**	**transferase activity, transferring phosphorus-containing groups**	**2.99E-4**	**13/53**
**GO:1901363**	**heterocyclic compound binding**	**3.22E-4**	**34/53**
**GO:0097159**	**organic cyclic compound binding**	**4.1E-4**	**34/53**
**GO:0003677**	**DNA binding**	**4.38E-4**	**21/53**
